# Supplementary material for: Dynalign II: common secondary structure prediction for RNA homologs with domain insertions
Source: Nucleic Acids Res. 2014 Nov 21;42(22):13939–48. doi: 10.1093/nar/gku1172 (PMC4267632; doi:10.1093/nar/gku1172)
Supplement: SUPPLEMENTARY DATA [file supp_gku1172_nar-02021-z-2014-File012.zip › manual/Text/Multifind.html]

RNAstructure Command Line Help: Multifind


|  |  |  |
| --- | --- | --- |
|  | RNAstructure Command Line Help Multifind | - Contents - Index |
| Multifind uses multilign, which predicts common structures for multiple homologous sequences, and estimates the probability that the input sequences are a conserved non-coding RNA using a classification support vector machine. It has two dsitinct executables, a serial program called Multifind and a parallelized program called Multifind-smp for use in shared memory enviroments. USAGE: Multifind <fasta file> <Multifind output file> <ct file 1> <ct file 2> <ct file 3>... [options] Required parameters: <fasta file> \| The name of a fasta file containing input alignment data. Note that lowercase nucleotides are forced single-stranded in structure prediction.; <Multifind output file> \| The name of a Multifind outpur file to which output will be written. It includes output alignment and probability of the sequences in the alignment input being conserved non-coding RNA inferred by SVM.; <ct file 1>, <ct file 2>, <ct file 3>... \| The names of ct files containing output structure data for all the sequences in the input alignment. A name needas to be specified for each sequence in the fasta file. Options which require added values: -p, -P, --processors \| Specify the number of processors/cores required for parallelized program Multifind-smp. This is only required for Multifind-smp. Default is 1. References: 1. Reuter, J.S. and Mathews, D.H. "RNAstructure: software for RNA secondary structure prediction and analysis." *BMC Bioinformatics*, 11:129. (2010). 2. Mathews, D.H., Disney, M.D., Childs, J.L., Schroeder, S.J., Zuker, M. and Turner, D.H. "Incorporating chemical modification constraints into a dynamic programming algorithm for prediction of RNA secondary structure." *Proc. Natl. Acad. Sci. USA*, 101:7287-7292. (2004). 3. Mathews, D.H., Sabina, J., Zuker, M. and Turner, D.H. "Expanded sequence dependence of thermodynamic parameters provides improved prediction of RNA secondary structure." *J. Mol. Biol.*, 288:911-940. (1999). | | |
| Visit The Mathews Lab RNAstructure Page for updates and latest information. | | |
